# Assessing the micro-scale environment using Google Street View: the Virtual Systematic Tool for Evaluating Pedestrian Streetscapes (Virtual-STEPS)

**DOI:** 10.1186/s12889-019-7460-3

**Published:** 2019-09-10

**Authors:** Madeleine Steinmetz-Wood, Kabisha Velauthapillai, Grace O’Brien, Nancy A. Ross

**Affiliations:** 1 Department of Geography, McGill University, 805 Sherbrooke St W, Montreal, QC H3A 0B9 Canada; 2 McGill School of Environment, McGill University, 805 Sherbrooke St W, Montreal, QC H3A 0B9 Canada

**Keywords:** Micro-scale, Built environment, Pedestrian, Audit, Google Street View, Virtual, Active living, Physical activity, Walkability

## Abstract

**Background:**

Altering micro-scale features of neighborhood walkability (e.g., benches, sidewalks, and cues of social disorganization or crime) could be a relatively cost-effective method of creating environments that are conducive to active living. Traditionally, measuring the micro-scale environment has required researchers to perform observational audits. Technological advances have led to the development of virtual audits as alternatives to observational field audits with the enviable properties of cost-efficiency from elimination of travel time and increased safety for auditors. This study examined the reliability of the Virtual Systematic Tool for Evaluating Pedestrian Streetscapes (Virtual-STEPS), a Google Street View-based auditing tool specifically designed to remotely assess micro-scale characteristics of the built environment.

**Methods:**

We created Virtual-STEPS, a tool with 40 items categorized into 6 domains (pedestrian infrastructure, traffic calming and streets, building characteristics, bicycling infrastructure, transit, and aesthetics). Items were selected based on their past abilities to predict active living and on their feasibility for a virtual auditing tool. Two raters performed virtual and field audits of street segments in Montreal neighborhoods stratified by the Walkscore that was used to determine the ‘walking-friendliness’ of a neighborhood. The reliability between virtual and field audits (*n* = 40), as well as inter-rater reliability (*n* = 60) were assessed using percent agreement, Cohen’s Kappa statistic, and the Intra-class Correlation Coefficient.

**Results:**

Virtual audits and field audits (excluding travel time) took similar amounts of time to perform (9.8 versus 8.2 min). Percentage agreement between virtual and field audits, and for inter-rater agreement was 80% or more for the majority of items included in the Virtual-STEPS tool. There was high reliability between virtual and field audits with Kappa and ICC statistics indicating that 20 out of 40 (50.0%) items had almost perfect agreement and 13 (32.5%) items had substantial agreement. Inter-rater reliability was also high with 17 items (42.5%) with almost perfect agreement and 11 (27.5%) items with substantial agreement.

**Conclusions:**

Virtual-STEPS is a reliable tool. Tools that measure the micro-scale environment are important because changing this environment could be a relatively cost-effective method of creating environments that are conducive to active living.

**Electronic supplementary material:**

The online version of this article (10.1186/s12889-019-7460-3) contains supplementary material, which is available to authorized users.

## Background

Evidence suggests that neighborhood built environments can support active living [1] and improve health outcomes [2, 3]. Most studies have examined how macro-scale elements of neighborhood walkability (connectivity, land-use mix, population density) contribute to active living and health. These findings can sometimes be difficult to implement in existing neighborhood settings. The street grid of North American cities is incredibly enduring and difficult to change [4] and changing macro-scale features can require substantial reconfiguration of the neighborhood layout.

Altering micro-scale features of neighborhood walkability (e.g., the presence and condition of benches, sidewalks, trees, crossing signals, walking paths, and cues of social disorganization or crime) is a relatively cost-effective and efficient method of creating environments that are conducive to active living [5]. Evidence suggests that micro-scale elements of the built environment can account for differences in walking behavior in neighborhoods with a similar macro-scale walkability and that changes to the micro-scale walkability of a place could potentially lead to substantial increases in walking behavior [6]. The evidence base on the contribution of micro-scale elements of the built environment to walking behavior is limited likely owing to the resource intensity of the traditional field-auditing approach. This approach requires auditors to be physically present to conduct audits, which can lead to considerable time and cost restraints even for very small-scale local studies [7, 8]. Technological advances have led to the development of virtual audits, efficient alternatives to observational field audits, that are safe for auditors require less time and financial resources, allow researchers to audit more study sites, and to use historical images to examine changes in built environments over time. They can also facilitate the auditing of dispersed, large, or distant areas [7, 9–11] improving the geographic scope and generalizability of findings, since variations in the built environment of neighborhoods implies that associations may also vary [11]. This method of data collection is also more flexible compared to in-person auditing methods, as auditors can easily refer to street stills at a later point in time if they discover that the assessment of additional environmental features is warranted.

Research has demonstrated that virtual audits can provide a valid alternative to field audits for measuring micro-scale features [12]. Zhu and colleagues [12] created an online version of the Microscale Audit of Pedestrian Streetscapes tool, a well-known field auditing tool developed by Millstein and colleagues [13], that was modified to render it compatible with virtual auditing [12]. The researchers audited designated routes in San Diego and Phoenix, and consistent with previous research, demonstrated a higher reliability for items that involve the verification of the presence of an item and lower reliability for items that are temporally variable or that require a subjective assessment [12]. This research is promising because it suggests that virtual audit tools can reliably assess the micro-scale environment.

Most published virtual audits have been performed with Google Street View (GSV), a web-service that has existed since 2007 (www.google.com/maps) [14]. Google, an American-based, international private technology company, best known for its internet search engine, continues to support broad non-commercial access to their mapping products for research and creative purposes (https://www.google.com/permissions/geoguidelines.html). GSV was originally only available in U. S cities but has progressively expanded to provide video stills of streets from across the world [15]. GSV can be accessed through Google Maps or Google Earth and provides a 360° horizontal and 290° vertical panoramic view of the streets. This tool has allowed researchers to perform audits at their desk by virtually “navigating” streetscapes.

Previous research has indicated high levels of agreement between virtual and field audits [10, 15–19] and has shown high agreement between GSV audits and assessments obtained from local residents [20]. Many previous virtual auditing tools have not been specifically designed to measure the micro-scale environment meaning that they aren’t designed to minimize the limitations associated with virtually auditing this environment. These tools are also often very lengthy (i.e., have many items), which means that even if street segments are evaluated virtually and travel time is eliminated, auditing is still a very time-consuming process and not practical for the assessment of large geographic areas.

### Objective

The objective of this study was to examine the reliability of the Virtual Systematic Tool for Evaluating Pedestrian Streetscapes (Virtual-STEPS), an auditing tool specifically designed to remotley evaluate micro-scale characteristics of the built environment. To achieve this aim, we examined the agreement between virtual and field audits and the inter-rater agreement for the Virtual-STEPS tool.

## Methods

In November 2017, 2200 adults in Montreal and Toronto were recruited as part of a study to examine the impact of the walkability of neighborhoods on active living. Participants were recruited from 136 (68 from Montreal/68 from Toronto) forward sortation areas (FSA) (first three digits of the postal code). On average, there are 8000 households within an FSA [21]. One of the aims of the study was to develop an auditing tool that could potentially be applied at a national level to identify the micro-scale environmental features that support walking in Canadian cities.

### Virtual Systematic Tool for Evaluating Pedestrian Streetscapes (Virtual-STEPS)

The Virtual Systematic Tool for Evaluating Pedestrian Streetscapes (Virtual-STEPS) is an observational audit tool that uses GSV to assess micro-scale features of neighborhood environments that might support active living. Two research assistants conducted a comprehensive literature review and identified 40 micro-scale elements of neighborhood environments that might support active living. The publications that influenced the creation of each item are included in Table 1. These items were categorized as follows: **pedestrian infrastructure** (e.g., sidewalks), **traffic calming and streets** (e.g., stop signs), **building characteristics** (e.g., length of building setback), **bicycling infrastructure** (e.g., bicycling lanes), **transit** (e.g., bus stops), and **aesthetics/disorder** (e.g., graffiti). The tool emphasized the inclusion of micro-scale features that have been found to contribute to active living in previous studies but are not usually readily available as Geographic Information System (GIS) layers in administrative databases, and those features that are feasible to measure using GSV (e.g., time provided to cross the street by a pedestrian signal might support walking but can’t be assessed in GSV).

**Table 1:** The 40 Virtual-STEPS tool items and their categories grouped into six domains

Items	Categories	Publications
Pedestrian Infrastructure		
Presence of a Sidewalk	Present-one side/Present-both sides/Not present	[13, 22–25]
Sidewalk Continuity	Yes/No	[13, 24, 26]
Sidewalk Buffer	Yes/No	[13, 14, 22–24, 26, 27]
Sidewalk Quality	Good quality/Bad quality	[13, 14, 22–29]
Pedestrian Signal/Timer	Yes/No	[13, 22–24, 26, 27]
Pedestrian Crossing Sign	Yes/No	[22, 24, 28, 29]
Crosswalk Markings	Yes/No	[13, 22, 24, 26, 27]
Benches	Yes/No	[6, 22, 25–28, 30, 31]
Streetlights	None/Some/Many	[13, 15, 16, 22, 24–28, 32]
Curb Cuts	Yes/No	[13, 15, 22–28]
Curb Cut Quality	Good quality/Bad quality	
Tactile Paving	Yes/No	[15, 24, 33]
Traffic Calming and Streets		
Traffic Lights	Yes/No	[13, 22–24, 26, 28, 29, 32]
Traffic Island	Yes/No	[22, 27–29, 32]
Stop Lines	Yes/No	
Stops Signs	Yes/No	[13, 22, 24, 26, 29]
Curb Extension	Yes/No	[13, 22–24, 28, 29]
Speed Bump	Yes/No	[22–24, 28, 29]
Bollards	Yes/No	[31]
Number of Traffic Lanes	Continuous	[13, 16, 23–29]
Number of Parking Lanes	Continuous	[16, 23, 24, 26, 27, 29]
Driveways	None/Some/Many	[16, 21, 22, 28]
Building Characteristics		
Building Height	N/A/1–2 stories/3–5 stories/6+ stories	[6, 13, 22, 28, 30]
Building Setback	N/A/0 m/0-3 m/3-10 m/> 10 m	[6, 13, 28, 32, 34]
Building Design Variation	N/A/None/Some/A lot	[13, 22, 23, 27, 28, 30, 34, 35]
Transit		
Presence of Transit	Yes/No	[13, 6, 22, 24–27, 29]
Type of Transit	Bus/Metro/Train	[33]
Transit Facilities	None/Bench or shelter/Both	[13, 28]
Bicycling Infrastructure		
Bike Lanes	Yes/No	[13, 22, 24, 25, 29, 32]
Bike Buffer	Yes/No	
Bike Facilities	Yes/No	[16, 23, 29]
Aesthetics/Disorder		
Trees	None/Few/Some/Many	[13, 15, 16, 22–27, 36]
Shade	< 30% of the street /≥30% of the street	[22, 27–29, 31]
Nature Areas	Yes/No	[22, 28, 30, 36]
Landscaping	None/Some/A lot	[13, 27, 31]
Landscape Maintenance	Yes/No	[6, 13, 23, 24, 27, 31, 33, 36]
Presence of Litter	None/Some/A lot	[22–27, 29]
Graffiti	None/Some/A lot	[13, 15, 16, 22–27, 29, 32]
Broken/Boarded Windows	Yes/No	[4, 13, 25, 27]
Attractive Segment	Unattractive/Neutral/Attractive	[16, 22, 23, 28]

### Street selection

**Fig. 1:**
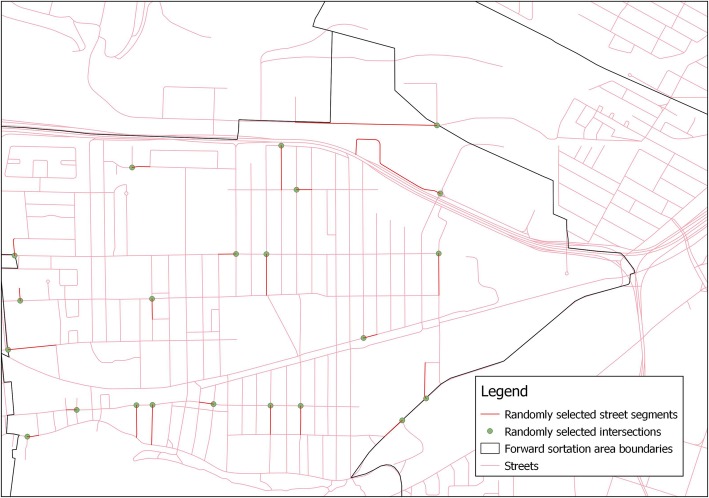
Randomly selected streets and randomly selected audit start points within a forward sortation area

The auditing of street segments occurred between June and September 2017, using street segments from the two largest cities in Canada - Toronto and Montreal. We tested the reliability of the tool using Montreal street segments for practical locational reasons. Street segments are sections of a street that are located between two neighboring intersections or an intersection and a cul-de-sac (Fig. 1). We randomly selected street segments from within Montreal forward sortation areas (first three digits of the postal code) based on known levels of neighborhood walkability. The walkability of forward sortation areas was measured by taking the average of the Walk Scores® associated with the 6-digit postal codes located within each forward sortation area. The Walk Score® is an index that determines the walkability of a location based on the distance between that location and different types of amenities (http://www.walkscore.com/methodology.shtml). Walk Score has been validated against other measures of walkability in previous studies [37, 38].

To test the agreement between virtual and field audits, virtual audits and field audits were conducted by one rater for 40 street segments (10 high walkability (Walkscore®: 70–89) /20 medium walkability (Walkscore®: 50–69) /10 low walkability (Walkscore®: 0–49)). When testing the agreement between virtual and field audits, the virtual audits of street segments from one rater were compared to the field audits of the same street segments conducted by the same rater (i.e., virtual audit of a street segment conducted by rater 1 was compared to the field audit of the same segment conducted by rater 1). To test inter-rater reliability, 60 of the same street segments were virtually evaluated by both raters (20 high walkability/20 medium walkability/20 low walkability). We stratified by walkability to ensure that there would be enough variability in the built environment in our sample of streets and to decrease the likelihood that we would have high percent agreement but low Kappa statistics due to low frequencies of features in the environment.

### Audit procedure

The two raters (KV and GO), that had contributed to the literature review and development of the Virtual-STEPS audit tool, conducted virtual and field audits of selected street segments. Raters travelled in person to street segments and conducted field audits independently. They also conducted virtual audits independently on separate computers. Field audits were conducted by walking down the street segment and auditing one intersection and both sides of the street. The start points (intersection) for the audits were selected randomly using Geographic Information Systems. The same auditing procedures were conducted for the virtual audits of street segments. To locate segments, we used QGIS, along with the go2streetview plugin (© 2014 Enrico Ferreguti). Auditors remotely audited the street segments using the most recent images available on GSV. For the virtual audits, raters also noted the year of the GSV images and whether their view was obstructed, or the image was distorted.

**Fig. 2:**
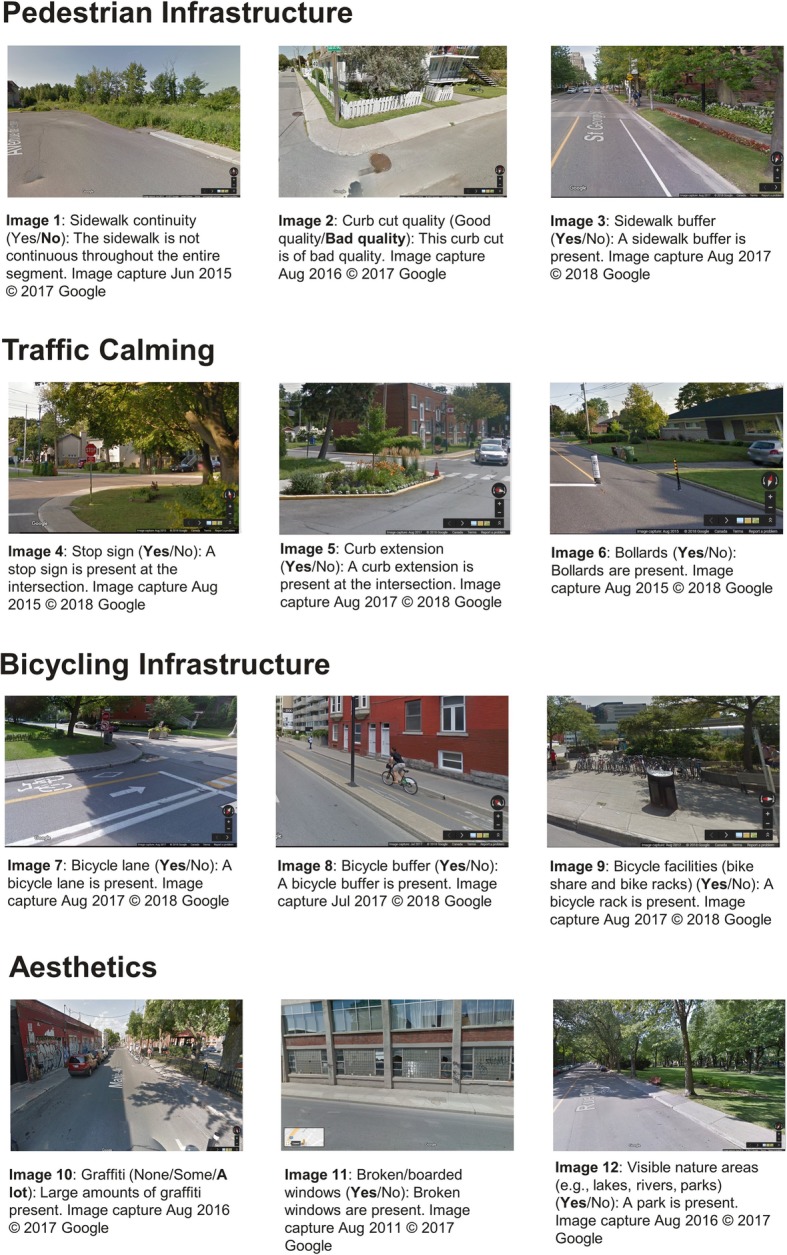
Examples of ratings for Virtual-STEPS items. Image captures from Google Street View (www.google.com/maps)

The audit process unfolded as follows: (1) The attribute table was opened and the segment was selected using ‘zoom to feature’; (2) Once the segment appeared in the QGIS map the go2streetview plugin was used to find the appropriate intersection in GSV; (3) The intersection and the segment were audited with results input into a Microsoft Access database. The auditing process involved an assessment of features belonging to both sides of the segment, as well as the given intersection. The items assessed at intersections were crossing aids, curb cuts, curb tactile paving, curb quality, and certain traffic calming devices (e.g., traffic lights, stop lines, stop signs, and traffic islands). Transit stops, benches, and bike facilities were assessed along the segment and at the intersection. Examples of ratings are included in Fig. 2.

It was important to ensure that it would be feasible to apply our auditing method across large geographic areas. To achieve this goal, we audited the first 300 m of each street segment. Previous studies have eliminated streets over 300 m from the dataset to ensure consistency [15]. We chose to include all segments over 300 m, but for segments over this length to only audit the first 300 m of the segment and the street segment was given the rating derived from the first 300 m. This approach allowed for the retention of longer streets in the database that might be important contributors to the overall micro-scale environment of a neighborhood. We also compared audits conducted on the first 300 m of streets segments to audits conducted with the entire street segment for 32 randomly selected streets over 300 m with an average length of 592.82 (SD:519.4). This comparison yielded an average percent agreement of 98% (see Additional file 1).

### Analysis

The reliability between GSV and field audits and the inter-rater reliability of observed audit characteristics was calculated using Cohen’s Kappa coefficient [39]. The Kappa coefficient accounts for agreement that would be expected to occur by chance (a value of 1 corresponds to perfect agreement and 0 corresponds to agreement that likely occurred by chance) [40]. Weighted Kappa was used for ordinal variables. Cohen’s Kappa coefficients have been classified into: < 0.20 (poor agreement), 0.21–0.40 (fair agreement), 0.41–0.60 (moderate agreement), 0.61–0.80 (substantial agreement), 0.81–1.00 (almost perfect agreement) [40]. Percent agreement was also reported due to the Cohen’s Kappa coefficient’s sensitivity to prevalence, which can lead to high absolute agreement but low Kappa [41]. The Intraclass Correlation Coefficient (ICC) was used for continuous variables and the same classification system was also used to interpret ICC values [13, 42]. Analyses were performed using R.

## Results

Forty street segments were evaluated for agreement between virtual and field audits. One street segment was removed due to image obstructions. Sixty street segments were evaluated for inter-rater reliability. One street segment was removed due to image obstructions. Virtual audits took, on average 9.8 (range:19) minutes per street segment, while field audits took approximately 8.2 (range:16) minutes plus travel time.

### Agreement between virtual and field audits

Absolute agreement was high with 32 of 40 (80.0%) items having an absolute agreement above 80%. The average absolute agreement was above 80% for **pedestrian infrastructure** (92.5%), **traffic calming and streets** (92.8%), **transit** (99.1%), **bicycling infrastructure** (94.0%), **building characteristics** (81.1%), and **aesthetics/disorder** (80.4%). Kappa and ICC statistics indicated that 20 of 40 (50.0%) items had almost perfect agreement (Kappa or ICC > 0.80), 13 (32.5%) items had substantial agreement (Kappa or ICC 0.61–0.80), 6 (15.0%) items had moderate agreement (Kappa or ICC 0.41–0.60), 1 (2.5%) item had fair agreement (Kappa or ICC 0.21–0.40), and that no items had poor agreement.

### Inter-rater reliability

Absolute agreement was high with 30 of 40 items (75%) with an absolute agreement above 80%. The average absolute agreement was above 80% for **pedestrian infrastructure** (93.5%), **traffic calming and streets** (91.0%), **transit** (98.3%), **bicycling infrastructure** (97.2%), **building characteristics** (82.4%), and was slightly lower for **aesthetics/disorder** (76.6%). Kappa and ICC statistics indicated that 17 items (42.5%) had almost perfect agreement (Kappa or ICC > 0.80), 11 (27.5%) items had substantial agreement (Kappa or ICC 0.61–0.80), 6 (15.0%) items had moderate agreement (Kappa or ICC 0.41–0.60), 2 (5.0%) items had fair agreement (Kappa or ICC 0.21–0.40), and 1 (2.5%) item had poor agreement (Kappa or ICC < 0.21). The item “shade” (i.e., “Is 30% of the street sheltered from the sun”) is included in Table 2 but will be removed from the tool due to an inter-rater reliability with both a poor percent agreement and low Kappa. For 3 items a Kappa coefficient couldn’t be calculated (broken/boarded windows, sidewalk buffer, and bollards) due to a low frequency (*n* = 0) in the streets selected for inter-rater reliability (See Additional file 2).

**Table 2:** Results for inter-rater reliability and reliability between GSV and in-field audits using percent agreement and the Kappa statistic

	GSV with field		Inter-rater	
Item	Percent agreement	Kappa or ICC	Percent agreement	Kappa or ICC
Pedestrian Infrastructure				
Presence of Sidewalks	100	1.00	96.6	0.97
Sidewalk Continuity	94.9	0.87	94.9	0.90
Sidewalk Buffer	100	1.00	100	N/A
Sidewalk Quality	82.1	0.63	91.5	0.81
Pedestrian Sign/Timer	100	1.00	100	1.00
Pedestrian Crossing Sign	92.3	0.63	94.9	0.38
Crosswalk Markings	92.3	0.85	96.6	0.91
Benches	89.7	0.73	94.9	0.74
Streetlights	69.2	0.51	78.0	0.69
Curb Cuts	97.4	0.93	91.5	0.83
Curb Cut Quality	94.9	0.64	93.2	0.31
Tactile Paving	97.4	0.93	89.8	0.79
Traffic Calming and Streets				
Traffic Lights	100	1.00	100	1.00
Traffic Island	97.4	0.84	94.9	0.80
Stop Lines	89.7	0.77	91.5	0.82
Stops Signs	97.4	0.98	96.6	0.91
Curb Extension	97.4	0.65	98.3	0.79
Speed Bump	97.4	0.66	98.3	0.66
Bollards	97.4	0.84	98.3	N/A
Number of Traffic Lanes	87.2	0.84	81.4	0.70
Number of Parking Lanes	76.9	0.82	66.1	0.64
Driveways	87.2	0.85	84.7	0.76
Building Characteristics				
Building Height	89.4	0.88	94.9	0.91
Building Setback	87.2	0.88	82.8	0.83
Building Design Variation	66.7	0.47	69.5	0.47
Transit				
Presence of Transit	100	1.00	98.3	0.91
Type of Transit	97.4	0.92	98.3	0.93
Transit Facilities	100	1.00	98.3	0.97
Bicycling Infrastructure				
Bike Lanes	92.3	0.75	98.3	0.91
Bike Buffer	100	1.00	100	1.00
Bike Facilities	89.7	0.71	93.2	0.63
Aesthetics				
Presence of Trees	76.9	0.70	61	0.55
Shade	79.5	0.55	49.2	0.16
Nature Areas	82.1	0.62	84.7	0.69
Landscaping	79.5	0.56	86.4	0.42
Landscape Maintenance	94.9	0.72	86.4	0.42
Presence of Litter	71.8	0.47	71.2	0.54
Graffiti	84.6	0.69	94.9	0.88
Broken/Boarded Windows	87.2	0.39	98.3	N/A
Attractive Segment	66.7	0.58	57.6	0.44

## Discussion

The Virtual- STEPS tool can provide a reliable measure of micro-scale characteristics that may support active living. Absolute agreement between virtual and field audits and inter-rater agreement was 80% or more for most items included in the Virtual-STEPS tool. Most items also had high to moderate levels of agreement according to Cohen’s Kappa coefficients.

Congruent with previous research [8, 12, 15], the tool demonstrated higher reliability between virtual and field audits and inter-rater reliability for items that involve the verification of the presence/absence of large items (e.g., presence of traffic calming features, transit facilities, bike lanes, and bike buffers). This may be due to the fact that these items can be easily spotted by car and car-based cameras are used to capture GSV images [7]. The tool had lower reliability for items that require a subjective evaluation of a neighborhood characteristic [8, 12, 15, 43] such as those that assess the condition of features (e.g., curb cut quality, landscape maintenance), variations in the environment (e.g., building design variation), or the aesthetics of the neighborhood (e.g., graffiti, litter, presence of landscaping, attractiveness of the segment). The temporal variability of certain aesthetic elements such as graffiti and litter could also explain the lower reliability observed between virtual audits and field audits for these items.

We chose to design a tool to specifically measure features of the micro-scale environment that may support active living, given the potential for these features to be reasonably modified within the scale of budgets of local governments. Transforming the micro-scale environment could have a meaningful impact on the active living potential of places [5, 6, 44, 45]. For example, Sallis and colleagues, showed that an increase from the lowest quintile of micro-scale walkability to the highest quintile might lead to an almost 250% increase in walking for transportation in younger and older adults [6]. Micro-scale features of the built environment that are unfavorable to active living may also actually offset the benefits of macro-scale walkability for vulnerable populations such as the elderly and the physically impaired [5, 46] contributing to the disproportionately high burden of poor urban design born by these population groups [47].

### Strengths and limitations

A lack of certain features in the environment can result in low Cohen’s Kappa values but high percent agreement [8]. We attempted to minimize this issue by including neighborhoods varying in neighborhood walkability (low/medium/high) in our assessment. Items with high percent agreement but for which a Kappa could not be calculated (e.g., bollards, broken/boarded windows) were retained in the tool because although the items did not occur frequently for the specific street segments selected, we still considered them to be important contributors to the walkability of neighborhoods. The item that asked the auditor to assess whether 30% or more of the segment was shaded from the sun had poor reliability that could not be explained by a low frequency in the selected street segments. This item was removed from the tool because although the benefit of including the item in the tool could be substantial, especially as heat events in cities are anticipated to rise, the inter-rater reliability was poor suggesting that raters had considerable difficulty agreeing on whether 30% of a street segment was shaded.

Virtual audits do not incorporate sensory inputs such as noise levels, soundscape, and scent [7] that may contribute to a pedestrians experience of a streetscape. GSV images may also change unpredictably. A previous study showed that this was common when virtually crossing intersections [9] leading to temporal inconsistencies in the year or season of the images used for audits [7]. The auditors identified several shortcomings to the use of GSV. Compared to field audits, it was difficult to evaluate finer details of streetscapes such as condition (e.g., quality of sidewalks and curb cuts) and maintenance (e.g., landscape maintenance). Further, although GSV does provide a good “street view” it does not always provide an accurate “pedestrian view”. GSV provides a view that is a bit higher than the typical pedestrian view with the images recorded from a car-mounted camera. The use of virtual audits with GSV therefore may result, for example, in the inclusion of features that will not necessarily influence the pedestrian experience such as including features on the other side of a large fence in micro-scale assessments when these features may not be visible by pedestrians.

Our results concur with the sentiments of Griew and colleagues who expressed that the advantages of virtual audits greatly outweigh their limitations [15]. GSV allowed auditors to comfortably and safely audit features that were more difficult or dangerous to audit in person such as the presence of broken/boarded windows. Another item that auditors had difficulty auditing in person was setback length. In contrast, in virtual audits, auditors could easily approximate average setback length using the measurement tool in Google Satellite.Virtual-STEPS takes less than 10 min to complete and contains 40 reliable items that cover a variety of concepts that have been demonstrated to influence walking in past research. The items are also highly reliable between raters and reliably reflect field audits. The virtual audits took slightly longer than in-field audits (excluding travel time) to conduct on average because auditors had to virtually ‘walk’ down the street more than once using different camera angles to assess different items. Despite a slightly longer average auditing time, virtual audits were still much less expensive and time-consuming to conduct compared to field audits because field audits require significant amounts of travel time.

Our study differentiates itself from previous studies that have evaluated the micro-scale environment remotely such as that of Zhu and colleagues [12] by creating a tool that is specifically designed to measure the micro-scale environment of large geographic areas. The tool responds to a need for auditing instruments that can efficiently be used for widespread surveillance. Existing auditing tools have an average of 92.2 items per tool [6, 13, 15, 22, 23, 26, 28, 34, 48] making it difficult to apply them for surveillance purposes. The Virtual-STEPS tool is user-friendly with only 40 items. We also included lengthy segments in our audits to ensure that all types of segments would be included in our sample, but only audited the first 300 m of each segment to maximize the tools potential for surveillance purposes. Our findings suggest that the tool has the potential to be used to assess the environments of large geographic areas and to be linked to large national scale administrative databases for epidemiological studies. This could enable the exploration of the variations in pedestrian streetscapes existing across cities and countries, subsequently allowing us to disentangle their contributions to active living across a diverse set of contexts. GSV Time-Machine could also allow the application of this auditing tool across images from multiple years allowing longitudinal examinations of changes in micro-scale environments that might be associated with health-related behavior changes. Machine learning techniques have been used with GSV to evaluate several characteristics of urban environments including pedestrian counts [49], visual enclosure [50], the construction and maintenance quality of building facades, and the continuity of the street wall [51]. The Virtual-STEPS tool was specifically designed for use with GSV giving it the potential to be used alongside and in validation of machine learning techniques for the automated extraction of built environment features for large scale surveillance.

## Conclusion

Our findings suggest that the Virtual-STEPS tool is a reliable tool for assessing the micro-scale environment of neighborhoods, potentially important contributors to active living and health. This tool can help researchers and public health practitioners to identify the routine micro-scale elements of the built environment that encourage active living. Elements that can be modified at relatively low cost to promote the mobility of the entire population, but could be especially valuable for the mobility of vulnerable populations such as the elderly and the physically impaired; populations that disproportionately bear the burden associated with sub-optimal urban design.

## Additional file

Additional file 1: Reliability of 300-meter segments with segments over 300 meters. (DOCX 16 KB)

Additional file 2: Prevalence of built environment features in the selected street segments. (DOCX 45 KB)

## Data Availability

The datasets used and/or analyzed during the current study are available from the corresponding author on reasonable request.

## Abbreviations

GSV: Google Street View
Virtual-STEPS: Virtual Systematic Tool for Evaluating Pedestrian Streetscapes
