# Supplementary material for: Assessing the micro-scale environment using Google Street View: the Virtual Systematic Tool for Evaluating Pedestrian Streetscapes (Virtual-STEPS)
Source: BMC Public Health. 2019 Sep 10;19:1246. doi: 10.1186/s12889-019-7460-3 (PMC6734502; doi:10.1186/s12889-019-7460-3)
Supplement: Supplementary file 1 — Reliability of 300-meter segments with segments over 300 meters. (DOCX 16 KB) [file 12889_2019_7460_MOESM1_ESM.docx]

Table 1: Reliability of 300-meter segments with segments over 300 meters.

|  | **300-meter with over 300 meter segments** | |  |
| --- | --- | --- | --- |
| **Item** | **Percent agreement** | **Kappa or ICC** |  |
| **Pedestrian Infrastructure** | |  |  |
| Presence of Sidewalks | 100 | 1.00 |  |
| Sidewalk Continuity | 100 | 1.00 |  |
| Sidewalk Buffer | 100 | 1.00 |  |
| Sidewalk Quality | 96.9 | 0.89 |  |
| Pedestrian Sign/Timer | 100 | 1.00 |  |
| Pedestrian Crossing Sign | 100 | 1.00 |  |
| Crosswalk Markings | 100 | 1.00 |  |
| Benches | 96.9 | 0.89 |  |
| Streetlights | 96.9 | 0.96 |  |
| Curb Cuts | 100 | 1.00 |  |
| Curb Cut Quality | 100 | 1.00 |  |
| Tactile Paving | 100 | 1.00 |  |
| **Traffic Calming and Streets** |  |  |  |
| Traffic Lights | 100 | 1.00 |  |
| Traffic Island | 100 | 1.00 |  |
| Stop Lines | 100 | 1.00 |  |
| Stops Signs | 100 | 1.00 |  |
| Curb Extension | 100 | N/A |  |
| Speed Bump | 100 | 1.00 |  |
| Bollards | 100 | N/A |  |
| Number of Traffic Lanes | 100 | 1.00 |  |
| Number of Parking Lanes | 100 | 1.00 |  |
| Driveways | 87.5 | 0.75 |  |
| **Building Characteristics** |  |  |  |
| Building Height | 93.8 | 0.85 |  |
| Building Setback | 90.6 | 0.80 |  |
| Building Design Variation | 90.6 | 0.803 |  |
| **Transit** |  |  |  |
| Presence of Transit | 100 | 1.00 |  |
| Type of Transit | 100 | 1.00 |  |
| Transit Facilities | 96.9 | 0.90 |  |
| **Bicycling Infrastructure** |  |  |  |
| Bike Lanes | 96.9 | 0.87 |  |
| Bike Buffer | 96.9 | 0.65 |  |
| Bike Facilities | 100 | 1.00 |  |
| **Aesthetics** |  |  |  |
| Presence of Trees | 84.4 | 0.71 |  |
| Shade | 96.9 | 0.94 |  |
| Nature Areas | 100 | 1.00 |  |
| Landscaping | 96.9 | 0.93 |  |
| Landscape Maintenance | 96.9 | 0.93 |  |
| Presence of Litter | 100 | 1.00 |  |
| Graffiti | 100 | 1.00 |  |
| Broken/Boarded Windows | 100 | 1.00 |  |
| Attractive Segment | 96.9 | 0.96 |  |
