# Supplementary material for: Assessing the micro-scale environment using Google Street View: the Virtual Systematic Tool for Evaluating Pedestrian Streetscapes (Virtual-STEPS)
Source: BMC Public Health. 2019 Sep 10;19:1246. doi: 10.1186/s12889-019-7460-3 (PMC6734502; doi:10.1186/s12889-019-7460-3)
Supplement: Supplementary file 2 — Prevalence of built environment features in the selected street segments. (DOCX 45 KB) [file 12889_2019_7460_MOESM2_ESM.docx]

Table 1: Prevalence of built environment features in the selected street segments.

| GSV vs field | | | | |  | Inter-rater | | | |
| --- | --- | --- | --- | --- | --- | --- | --- | --- | --- |
| GSV | | % or mean(SD) | Field | % or mean(SD) |  | Rater 1 | % or mean(SD) | Rater 2 | % or mean(SD) |
| **Pedestrian Infrastructure** | |  |  |  |  |  |  |  |  |
| Presence of Sidewalks | | |  |  |  |  |  |  |  |
| Absent | 7 | 17.95 | 7 | 17.95 |  | 28 | 47.46 | 27 | 45.76 |
| Present - 1 side | 3 | 7.69 | 3 | 7.69 |  | 6 | 10.17 | 6 | 10.17 |
| Present - Both sides | 29 | 74.36 | 29 | 74.36 |  | 25 | 42.37 | 26 | 44.07 |
| Sidewalk Continuity | | |  |  |  |  |  |  |  |
| Absent | 10 | 25.64 | 12 | 30.77 |  | 30 | 50.85 | 33 | 55.93 |
| Present | 29 | 74.36 | 27 | 69.23 |  | 29 | 49.15 | 26 | 44.07 |
| Sidewalk Buffer | |  |  |  |  |  |  |  |  |
| Absent | 38 | 97.44 | 38 | 97.44 |  | 59 | 100 | 59 | 100 |
| Present | 1 | 2.56 | 1 | 2.56 |  | 0 | 0 | 0 | 0 |
| Sidewalk Maintenance | | |  |  |  |  |  |  |  |
| Absent | 23 | 58.97 | 22 | 56.41 |  | 39 | 66.1 | 38 | 64.41 |
| Present | 16 | 41.03 | 17 | 43.59 |  | 20 | 33.9 | 21 | 35.59 |
| Pedestrian Sign/Timer | | |  |  |  |  |  |  |  |
| Absent | 34 | 87.18 | 34 | 87.18 |  | 57 | 96.6 | 57 | 96.6 |
| Present | 5 | 12.82 | 5 | 12.82 |  | 2 | 3.3 | 2 | 3.3 |
| Pedestrian Crossing Sign | |  |  |  |  |  |  |  |  |
| Absent | 36 | 92.31 | 33 | 84.62 |  | 55 | 93.2 | 58 | 98.3 |
| Present | 3 | 7.69 | 6 | 15.38 |  | 4 | 6.78 | 1 | 1.69 |
| Crosswalk Markings | | |  |  |  |  |  |  |  |
| Absent | 20 | 51.28 | 19 | 48.72 |  | 46 | 77.97 | 44 | 74.58 |
| Present | 19 | 48.72 | 20 | 51.28 |  | 13 | 22.03 | 15 | 25.42 |
| Benches |  |  |  |  |  |  |  |  |  |
| Absent | 29 | 74.36 | 29 | 74.36 |  | 53 | 10.17 | 52 | 11.86 |
| Present | 10 | 25.64 | 10 | 25.64 |  | 6 | 89.83 | 7 | 88.14 |
| Streetlights | |  |  |  |  |  |  |  |  |
| None | 3 | 7.69 | 1 | 2.56 |  | 9 | 15.25 | 8 | 13.56 |
| Some | 22 | 56.41 | 14 | 35.9 |  | 25 | 42.37 | 32 | 54.24 |
| Many | 14 | 35.9 | 24 | 61.54 |  | 25 | 42.37 | 19 | 32.2 |
| Curb Cuts |  |  |  |  |  |  |  |  |  |
| Absent | 9 | 23.08 | 8 | 20.51 |  | 28 | 52.54 | 31 | 47.46 |
| Present | 30 | 76.92 | 31 | 79.49 |  | 31 | 47.46 | 28 | 52.54 |
| Curb Cut Quality | |  |  |  |  |  |  |  |  |
| Poor | 37 | 94.87 | 35 | 89.74 |  | 54 | 91.53 | 58 | 98.31 |
| Good | 2 | 5.13 | 4 | 10.26 |  | 5 | 8.47 | 1 | 1.69 |
| Tactile Paving | |  |  |  |  |  |  |  |  |
| Absent | 10 | 25.64 | 9 | 23.08 |  | 37 | 37.29 | 31 | 47.46 |
| Present | 29 | 74.36 | 30 | 76.92 |  | 22 | 62.71 | 28 | 52.54 |
| **Traffic Calming** | |  |  |  |  |  |  |  |  |
| Traffic Lights |  |  |  |  |  |  |  |  |  |
| Absent | 32 | 82.05 | 32 | 82.05 |  | 54 | 91.5 | 54 | 91.5 |
| Present | 7 | 17.95 | 7 | 17.95 |  | 5 | 8.5 | 5 | 8.5 |
| Traffic Island | |  |  |  |  |  |  |  |  |
| Absent | 35 | 89.74 | 36 | 92.31 |  | 49 | 16.95 | 52 | 11.86 |
| Present | 4 | 10.26 | 3 | 7.69 |  | 10 | 83.05 | 7 | 88.14 |
| Stop Lines |  |  |  |  |  |  |  |  |  |
| Absent | 13 | 33.33 | 13 | 33.33 |  | 37 | 37.29 | 36 | 38.98 |
| Present | 26 | 66.67 | 26 | 66.67 |  | 22 | 62.71 | 23 | 61.02 |
| Stops Signs | |  |  |  |  |  |  |  |  |
| Absent | 14 | 35.9 | 14 | 35.9 |  | 14 | 23.72 | 16 | 27.12 |
| Present | 25 | 64.1 | 25 | 64.1 |  | 45 | 76.27 | 43 | 72.88 |
| Curb Extension | |  |  |  |  |  |  |  |  |
| Absent | 38 | 97.44 | 37 | 94.87 |  | 57 | 96.61 | 58 | 98.31 |
| Present | 1 | 2.56 | 2 | 5.13 |  | 2 | 3.39 | 1 | 1.69 |
| Speed Bump | |  |  |  |  |  |  |  |  |
| Absent | 38 | 97.44 | 37 | 94.87 |  | 58 | 98.31 | 57 | 96.61 |
| Present | 1 | 2.56 | 2 | 5.13 |  | 1 | 1.69 | 2 | 3.39 |
| Bollards |  |  |  |  |  |  |  |  |  |
| Absent | 36 | 92.31 | 35 | 89.74 |  | 58 | 98.31 | 59 | 100 |
| Present | 3 | 7.69 | 4 | 10.26 |  | 1 | 1.69 | 0 | 0 |
| Driveways |  |  |  |  |  |  |  |  |  |
| None | 9 | 23.08 | 8 | 20.51 |  | 8 | 13.56 | 6 | 10.17 |
| Some | 14 | 35.9 | 13 | 33.33 |  | 21 | 35.59 | 21 | 35.59 |
| Many | 16 | 41.03 | 18 | 46.15 |  | 30 | 50.85 | 32 | 54.24 |
| Number of Traffic Lanes | 39 | 1.92(0.81) | 39 | 1.92 (0.81) |  | 59 | 2.00(0.59) | 59 | 2.03(0.67) |
| Number of Parking Lanes | 39 | 1.15(0.74) | 39 | 1.03(0.84) |  | 59 | 1.10(0.82) | 59 | 0.93(0.72) |
| **Building Characteristics** | | |  |  |  |  |  |  |  |
| Building Height | |  |  |  |  |  |  |  |  |
| N/A | 1 | 2.56 | 2 | 5.13 |  | 3 | 5.08 | 3 | 5.08 |
| 1-2 Stories | 17 | 43.59 | 16 | 41.03 |  | 42 | 71.19 | 40 | 67.8 |
| 3-5 Stories | 14 | 35.9 | 15 | 38.46 |  | 11 | 18.64 | 14 | 23.73 |
| 6 + Stories | 7 | 17.95 | 6 | 15.38 |  | 3 | 5.08 | 2 | 3.39 |
| Building Setback | |  |  |  |  |  |  |  |  |
| N/A | 2 | 5.13 | 2 | 5.13 |  | 3 | 5.08 | 3 | 5.08 |
| 0m | 4 | 10.26 | 3 | 7.69 |  | 7 | 11.86 | 5 | 8.47 |
| 0-3m | 6 | 15.38 | 7 | 17.95 |  | 2 | 3.39 | 7 | 11.86 |
| 3-10m | 19 | 48.72 | 19 | 48.72 |  | 29 | 49.15 | 26 | 44.07 |
| >10m | 8 | 20.51 | 8 | 20.51 |  | 17 | 28.81 | 18 | 30.51 |
| Building Design Variation | |  |  |  |  |  |  |  |  |
| N/A | 1 | 2.56 | 2 | 5.13 |  | 3 | 5.08 | 3 | 5.08 |
| None | 5 | 12.82 | 5 | 12.82 |  | 0 | 0 | 3 | 5.08 |
| Some | 25 | 64.1 | 24 | 61.54 |  | 47 | 79.66 | 37 | 62.71 |
| A lot | 8 | 20.51 | 8 | 20.51 |  | 9 | 15.25 | 16 | 27.12 |
| **Transit** |  |  |  |  |  |  |  |  |  |
| Presence of Transit | | |  |  |  |  |  |  |  |
| Absent | 32 | 82.05 | 32 | 82.05 |  | 53 | 10.17 | 52 | 11.86 |
| Present | 7 | 17.95 | 7 | 17.95 |  | 6 | 89.83 | 7 | 88.14 |
| Type of Transit | |  |  |  |  |  |  |  |  |
| N/A | 32 | 82.05 | 32 | 82.05 |  | 53 | 89.83 | 52 | 88.14 |
| Bus/tram | 6 | 15.38 | 7 | 17.95 |  | 5 | 8.47 | 6 | 10.17 |
| Metro | 1 | 2.56 | 0 | 0 |  | 1 | 1.69 | 1 | 1.69 |
| Train | 0 | 0 | 0 | 0 |  | 0 | 0 | 0 | 0 |
| Transit Facilities | |  |  |  |  |  |  |  |  |
| N/A | 32 | 82.05 | 32 | 82.05 |  | 53 | 89.83 | 52 | 88.14 |
| Bench or shelter | 2 | 5.13 | 2 | 5.13 |  | 1 | 1.69 | 1 | 1.69 |
| Both | 3 | 7.69 | 3 | 7.69 |  | 3 | 5.08 | 3 | 5.08 |
| None | 2 | 5.13 | 2 | 5.13 |  | 2 | 3.39 | 3 | 5.08 |
| **Bicycling Infrastructure** | | |  |  |  |  |  |  |  |
| Bike Lanes | |  |  |  |  |  |  |  |  |
| Absent | 32 | 82.05 | 31 | 79.49 |  | 53 | 89.83 | 52 | 88.14 |
| Present | 7 | 17.95 | 8 | 20.51 |  | 6 | 10.17 | 7 | 11.86 |
| Bike Buffer |  |  |  |  |  |  |  |  |  |
| Absent | 38 | 97.44 | 38 | 97.44 |  | 58 | 98.31 | 58 | 98.31 |
| Present | 1 | 2.56 | 1 | 2.56 |  | 1 | 1.69 | 1 | 1.69 |
| Bike Facilities | |  |  |  |  |  |  |  |  |
| Absent | 30 | 76.92 | 30 | 76.92 |  | 53 | 89.83 | 53 | 89.83 |
| Present | 9 | 23.08 | 9 | 23.08 |  | 6 | 10.17 | 6 | 10.17 |
| **Aesthetics** | |  |  |  |  |  |  |  |  |
| Presence of Trees | |  |  |  |  |  |  |  |  |
| None | 1 | 2.56 | 1 | 2.56 |  | 3 | 5.08 | 5 | 8.47 |
| Few | 8 | 20.51 | 7 | 17.95 |  | 6 | 10.17 | 3 | 5.08 |
| Some | 22 | 56.41 | 19 | 48.72 |  | 17 | 28.81 | 35 | 59.32 |
| Many | 8 | 20.51 | 12 | 30.77 |  | 33 | 55.93 | 14 | 23.73 |
| Shade |  |  |  |  |  |  |  |  |  |
| Absent | 25 | 64.1 | 25 | 64.1 |  | 12 | 79.66 | 40 | 32.2 |
| Present | 14 | 35.9 | 14 | 35.9 |  | 47 | 20.34 | 19 | 32.2 |
| Nature Areas | |  |  |  |  |  |  |  |  |
| Absent | 27 | 69.23 | 22 | 56.41 |  | 35 | 59.32 | 34 | 57.63 |
| Present | 12 | 30.77 | 17 | 43.59 |  | 24 | 40.68 | 25 | 42.37 |
| Softscape Features | |  |  |  |  |  |  |  |  |
| None | 1 | 2.56 | 0 | 0 |  | 0 | 0 | 0 | 0 |
| Some | 13 | 33.33 | 19 | 48.72 |  | 21 | 35.59 | 32 | 54.24 |
| Many | 25 | 64.1 | 20 | 51.28 |  | 38 | 64.41 | 27 | 45.76 |
| Softscape Maintenance | | |  |  |  |  |  |  |  |
| Absent | 5 | 12.82 | 3 | 7.69 |  | 7 | 11.86 | 9 | 15.25 |
| Present | 34 | 87.18 | 36 | 92.31 |  | 52 | 88.14 | 50 | 84.75 |
| Presence of Litter | |  |  |  |  |  |  |  |  |
| None | 13 | 33.33 | 12 | 30.77 |  | 25 | 42.37 | 18 | 30.51 |
| Some | 24 | 61.54 | 25 | 64.1 |  | 27 | 45.76 | 38 | 64.41 |
| Many | 2 | 5.13 | 2 | 5.13 |  | 7 | 11.86 | 3 | 5.08 |
| Graffiti |  |  |  |  |  |  |  |  |  |
| None | 28 | 71.79 | 24 | 61.54 |  | 45 | 76.27 | 47 | 79.66 |
| Some | 9 | 23.08 | 12 | 30.77 |  | 11 | 18.64 | 10 | 16.95 |
| A lot | 2 | 5.13 | 3 | 7.69 |  | 3 | 5.08 | 2 | 3.39 |
| Broken/Boarded Windows | |  |  |  |  |  |  |  |  |
| Absent | 37 | 94.87 | 32 | 82.05 |  | 58 | 98.31 | 59 | 100 |
| Present | 2 | 5.13 | 7 | 17.95 |  | 1 | 1.69 | 0 | 0 |
| Attractive Segment | | |  |  |  |  |  |  |  |
| Unattractive | 11 | 28.21 | 12 | 30.77 |  | 13 | 22.03 | 11 | 18.64 |
| Neutral | 9 | 23.08 | 12 | 30.77 |  | 24 | 40.68 | 12 | 20.34 |
| Attractive 19 | | 48.72 | 15 | 38.46 |  | 22 | 37.29 | 36 | 61.02 |
